# Student Acceptance of Digital Entrustable Professional Activities: Protocol for a Cohort Study

**DOI:** 10.2196/59326

**Published:** 2025-03-25

**Authors:** Maximilian Domann, Constanze Richters, Matthias Stadler

**Affiliations:** 1 Institute of Medical Education LMU University Hospital Ludwig Maximilians University Munich Munich Germany

**Keywords:** medical education, entrustable professional activities, EPAs, digital EPAs, technology acceptance model

## Abstract

**Background:**

Integrating digital entrustable professional activities (EPAs) and simulations in medical education represents a substantial shift toward competency-based learning. This approach focuses on developing specific skills through manageable units and enhancing proficiency in high-stakes environments. The technology acceptance model provides a framework to evaluate the adoption of these educational technologies, emphasizing the roles of perceived usefulness and ease of use.

**Objective:**

This cohort study aims to investigate the acceptance of digital EPAs among medical students within simulated training environments. It seeks to understand how perceived usefulness and ease of use influence this acceptance, guided by the principles of the technology acceptance model.

**Methods:**

The cohort study will involve medical students in the clinical phase of their education at Ludwig Maximilians University Munich. The survey, distributed through the Module-6 distributor, will capture their perceptions of digital EPAs. The data will be analyzed using regression analysis.

**Results:**

Data collection is anticipated to be complete by April 2025, with analysis concluded by May 2025. The results will provide insights into students’ attitudes toward digital EPAs and their willingness to integrate these tools into their learning.

**Conclusions:**

This study will contribute to the understanding of digital EPAs’ role in medical education, potentially guiding future design and implementation of these tools. While highlighting the importance of perceived usefulness and ease of use, the study also acknowledges limitations in sample size and recruitment methodology, indicating the need for further research with more diverse and larger groups. This research is poised to shape future medical training programs, aligning with the evolving landscape of medical education.

**International Registered Report Identifier (IRRID):**

PRR1-10.2196/59326

## Introduction

### Background

The concept of entrustable professional activities (EPAs) has changed the approach to medical education, shifting the focus toward competency-based learning. They cover core functions like educational theory, curriculum design, and assessment while also addressing emerging areas such as self-development and technology use in health care education [1]. EPAs are designed to break down complex medical skills into manageable units, allowing learners to gain proficiency in specific tasks progressively [1]. This methodology has been instrumental in ensuring that medical trainees are adequately prepared for the responsibilities they will face in their professional lives while empowering teaching staff to maximize the learning outcomes of their students [2].

Complementing EPAs, the use of simulations in medical training has become increasingly important. These simulations provide realistic, interactive environments where students can practice and hone their skills without the risks associated with real-life clinical settings. As highlighted by Chernikova et al [3] and Smetana and Bell [4], computer-based simulations, in particular, offer dynamic representations of real-world medical scenarios. These tools are invaluable for developing both the cognitive decision-making and motor skills essential in the medical field.

However, despite the advancements in and the separate explorations of EPAs and simulations, the combination of these two into a digital format, more specifically digital EPAs, remains largely unexplored. While both EPAs and simulations have been part of extensive research efforts individually, their integration into a digital EPA format offers a new development for these two teaching methods [1,3,4]. Yet digital EPAs and their acceptance among learners and educators remains largely unexamined [5].

In understanding the adoption and effectiveness of these educational technologies, the technology acceptance model (TAM) offers valuable insights. Introduced by Davies [6] and further explored by Marangunić and Granić [5], the TAM posits that perceived ease of use and usefulness are critical factors influencing the acceptance and integration of new technologies in learning environments. This model has been widely applied in various contexts to understand how users come to accept and use technological tools in their work.

The aim of this study is therefore to identify how well students perceive the usefulness of digital EPAs as well as their ease of use. In addition, we will investigate whether this perception influences students’ intention of using digital EPAs in their studies.

### EPAs in Medical Education

EPAs, as a didactic concept, focus on competency-based curricula to help students acquire specific competencies, potentially contributing to a more flexible duration of training [1]. They are characterized by a gradual reduction of supervision until learners can perform tasks completely independently [7]. This development occurs over five stages, starting with mere presence and observation to the independent guidance of younger learners [1,8]. EPAs represent specific professional activities distinguishing them from conventional learning objectives [2]. A competency-oriented teaching method could reduce the training duration for faster-learning medical students without compromising the quality of education [9]. Such a system could benefit not only medical students but also the teaching staff, program developers, and medical institutions, as well as other employees, by clearly defining which tasks can be performed with what degree of autonomy [1,2,10].

### Digital EPAs

Simulations, as interactive representations of real-world scenarios, play a crucial role in the acquisition of complex skills. Simulation-based learning, as demonstrated by Chernikova et al [3] in their 2020 meta-analysis, is a highly effective approach to designing learning environments in higher education and can be beneficial from the beginning of study programs for students at all levels. Simulations provide an effective medium for developing complex skills, such as those required in surgery, combining theoretical knowledge with practical expertise, such as seen in spinal surgery [11]. In combination with the concept of EPAs, as gradually increasing levels of clearly defined competencies, this suggests complementing real-life training with training in simulations. These simulations would need to precede real-life training by allowing students to practice the same competencies (digital EPAs). Systematic use of these digital EPAs could potentially substantially increase the quality of and reduce the time required for real-life training, which is often cost-intensive or requires rare and critical situations (eg, high-stakes operations).

However, such a shift in education would need to be accepted by all stakeholders (ie, medical educators, medical students, and patients).

### The TAM

The TAM has significantly influenced the understanding of technology adoption and use [5]. This model’s foundation was built on two critical factors: perceived usefulness and perceived ease of use [5]. These factors are pivotal in shaping users’ attitudes and intentions toward adopting technology. Davies’ [6] original model proposed that users’ motivation to use a system is influenced by these two beliefs [5]. Perceived usefulness reflects the degree to which a person believes that using a specific system would enhance their job performance [12]. Perceived ease of use refers to the belief that using a particular system would be effortless [12]. The interaction between these two beliefs plays a critical role in determining the user’s attitude toward the system and, consequently, their actual use behavior [6].

The significance of the TAM extends beyond traditional technologies and has proven to be indispensable, especially in designing user-friendly and effective technology-based learning systems. In a world where digital technologies are increasingly coming to the forefront, TAM remains a critical framework for researchers and practitioners to predict and understand user behavior toward new technological advancements.

### This Study

In this cohort study, we will focus on medical students, investigating how well they perceive the usefulness of digital EPAs as well as their ease of use in medical education. In addition, we will investigate whether this perception influences students’ intention of using digital EPAs in their studies. Since this teaching concept is largely unfamiliar to students, they are first presented with a written EPA, followed by a digital version, which the questionnaire will refer to. The example EPA, as well as the digital EPA, can be accessed via Open Science Framework (OSF) [13].

## Methods

### Recruitment Plans and Study Population

The study population for this cohort study will consist of medical students in the clinical phase of their studies. This cohort is optimally suited for the application of digital EPAs, as these are aimed at learning specific skills, which is the core of the clinical phase of medical studies. The plan is to distribute the survey link through the major study distribution lists of Ludwig Maximilians University Munich, such as the Module-6 distributor. This would ensure that only students in the clinical phase participate in the survey. In addition, students would have the flexibility to complete the questionnaire at their convenience.

### Inclusion Criteria

Medical students must be at least in their third year of medical training. This ensures that the preclinical foundational subjects, such as macroscopic and microscopic anatomy, physiology, biochemistry, and medical psychology, have already been completed. In addition, they must have passed their first medical state examination.

### Exclusion Criteria

Reasonable command of the German language is required to participate in this study.

### Data Analysis Plans

A regression analysis will be performed assuming a moderate effect size. Furthermore, an α level, common in social sciences, of .05 was chosen. The statistical power of the study was set standardly at *P*=.80. To calculate the necessary sample size for these specific parameters, the software G*Power 3.1 [14] was used. This calculation indicated that a sample size of 68 participants is required to meet the above criteria. To provide additional security and account for possible dropouts, a margin of 10% will be added to the calculated sample size. This means that a total of 75 participants should be recruited for the study. Assuming a response rate of approximately 25% (based on previous experiences), we will contact 300 students (about 25% of all viable students at Ludwig Maximilians University Munich) via email.

### Questionnaire and Hypotheses

#### Overview

The basis for the conception of the questionnaire is the research work by Abdul Ghani et al [15], which used the TAM as a foundation for evaluating digital game-based learning. The questionnaire was only adapted to the context of digital EPAs. The entire questionnaire can be found on OSF [13]. Both the German translation shown to the participants and an English version of the questionnaire can be found there. The hypotheses below were formulated based on the TAM and are to be explored through the questionnaire.

#### Construct: Perceived Usefulness

##### Operational Definition

Perceived usefulness [6] reflects students’ perception of whether the use of digital EPAs in a simulated environment will enhance their performance (independent variable).

##### Hypothesis

The more useful medical students find digital EPAs in medical simulations, the higher their behavioral intention to use this technology as part of their studies will be.

#### Construct: Perceived Ease of Use

##### Operational Definition

Perceived ease of use [6] refers to a student’s perception that using digital EPAs for learning skills during their medical studies will require minimal effort (independent variable).

##### Hypothesis

The easier medical students find the operation of digital EPAs in medical simulations to be, the greater their behavioral intention to use this technology will be.

#### Construct: Attitude

##### Operational Definition

Attitude [6] refers to student’s judgment on whether the use of digital EPAs is beneficial to them (independent variable).

##### Hypothesis

The more positive the attitude of medical students toward digital EPAs in medical simulations is, the higher their behavioral intention to use this technology will be.

#### Construct: Behavioral Intention (Operational Definition)

Behavioral intention [6] refers to a student’s intention to theoretically use digital EPAs for their studies during the clinical phase of medical school if they were to be offered.

Due to the construct of the TAM, “Behavioral Intention” is highly influenced by “Perceived Usefulness” and “Perceived Ease of Use,” making it the dependent variable.

### Digital EPAs

Due to the unfamiliar teaching concept of EPAs for students, as well as the digital EPAs, a formulated EPA and its digital counterpart were created based on a paper by Ten Cate and Taylor [1]. The digital EPA merely represents a theoretical implementation of the previously formulated EPA for students in the future. It cannot yet be tested digitally in this form and can, therefore, only be presented to students conceptually in the form of continuous text. The complete description of the EPA and the digital EPA can be found on OSF [13]. Additionally, there is both a German and an English version for the same reasons as with the above questionnaire.

### Ethical Considerations

Ethics approval by the host university is pending. The study will only be conducted once it is granted.

## Results

The data collection of this study is expected to be completed by the end of April 2025. Following this, data analysis is to take place, which is to be completed by May 2025, following the reporting standards of the *Journal of Medical Internet Research* for statistics.

All data, in anonymized form, and the code for analysis will be uploaded to the project’s OSF repository.

## Discussion

This study hypothesized that the higher students’ PU and PEU are, the higher their behavioral intention to use will be and that this relationship would lead to higher actual system use [6].

The basis for our study is, on the one hand, the increasing emergence of EPAs in medical curricula as well as in specialist training and continuing education and, on the other hand, the widespread use of digital simulations [1,3,4]. The merging of both concepts remains relatively unexplored and can only be considered a successful development of traditional EPAs if this concept is also accepted by the learners themselves. Using the tried and tested TAM model, a meaningful evaluation of this situation will be made [5]. Since digital EPAs can increasingly contribute to medical training improvement, our findings are to be considered as a starting point for the design and implementation of these didactic tools so that more effective implementation and more engaged use among learners can be promoted in the future.

Nevertheless, our study must be viewed in light of some limitations. The sample size and the methodology of recruitment could limit the generalizability of the results. Future studies should involve larger and more diverse groups of medical students to gain a more comprehensive understanding of acceptance factors. In addition, investigating the long-term effects of digital EPAs on learning outcomes could provide valuable insights.

In summary, this study should serve as a starting point for more extensive research projects so that factors influencing the acceptance of digital teaching technologies in medicine, particularly digital EPAs, can be comprehensively investigated. By emphasizing the importance of usefulness and ease of use for the acceptance and use of digital EPAs, our study opens new perspectives for the design of effective and engaging medical training programs that meet both the needs of the students and the requirements of a constantly changing medical training landscape.

## Abbreviations

EPA: entrustable professional activity
OSF: Open Science Framework
TAM: technology acceptance model

## Data Availability

All data will be made available in anonymized form on the Open Science Framework repository after completion of the study.
